# Sumatriptan‐naproxen sodium in migraine: A review

**DOI:** 10.1111/ene.16434

**Published:** 2024-09-24

**Authors:** Robyn‐Jenia Wilcha, Shazia K. Afridi, Piero Barbanti, Hans Christoph Diener, Tim Patrick Jürgens, Michel Lanteri‐Minet, Christian Lucas, Jerôme Mawet, Xavier Moisset, Antonio Russo, Simona Sacco, Alexandra J. Sinclair, Marja‐Liisa Sumelahti, Cristina Tassorelli, Peter J. Goadsby

**Affiliations:** ^1^ Headache Group, NIHR King's Clinical Research Facility and SLaM Biomedical Research Centre, The Wolfson Sensory, Pain and Regeneration Research Centre, Institute of Psychiatry, Psychology and Neuroscience King's College London London UK; ^2^ Neurology Department Guy's and St Thomas' NHS Foundation Trust London UK; ^3^ Headache and Pain Unit IRCCS San Raffaele Roma Rome Italy; ^4^ San Raffaele University Rome Italy; ^5^ Department of Neuroepidemiology, Institute for Medical Informatics, Biometry and Epidemiology (IMIBE) Medical Faculty of the University Duisburg‐Essen Essen Germany; ^6^ Neurologisches Zentrum Neurologische Klinik, KMG Klinikum Güstrow Güstrow Germany; ^7^ Klinik und Poliklinik für Neurologie Kopfschmerzzentrum Nord‐Ost Rostock Germany; ^8^ Pain Départment CHU Nice and FHU InovPain Université Côte Azur Nice France; ^9^ Inserm U1107, Neuro‐Dol, Trigeminal Pain and Migraine Université Clermont Auvergne Clermont‐Ferrand France; ^10^ Pain Clinic, Service de Neurochirurgie, Hôpital Salengro CHU de Lille Lille France; ^11^ Emergency Headache Centre, Department of Neurology (J.M.) Lariboisiere Hospital, Assistance Publique des Hopitaux de Paris Paris France; ^12^ CHU de Clermont‐Ferrand, Inserm, Neuro‐Dol, service de neurologie Université Clermont‐Auvergne Clermont‐Ferrand France; ^13^ Department of Advanced Medical and Surgical Sciences, Headache Centre University of Campania “Luigi Vanvitelli” Naples Italy; ^14^ Department of Biotechnological and Applied Clinical Sciences University of L'Aquila L'Aquila Italy; ^15^ Institute of Metabolism and Systems Research University of Birmingham Birmingham UK; ^16^ Department of Neurology, Queen Elizabeth Hospital Birmingham University Hospitals Birmingham NHS Foundation Trust Birmingham UK; ^17^ Faculty of Medicine and Health Technology University of Tampere Tampere Finland; ^18^ Department of Brain and Behavioral Sciences University of Pavia Pavia Italy; ^19^ Headache Science & Neurorehabilitation Center IRCCS Mondino Foundation Pavia Italy; ^20^ Department of Neurology University of California Los Angeles California USA

**Keywords:** acute migraine attack, combination tablet, pharmacokinetics, sumatriptan‐naproxen sodium, treatment

## Abstract

**Background:**

Varied responses to acute migraine medications have been observed, with over one‐third (34.5%) of patients reporting insufficient headache relief. Sumatriptan‐naproxen sodium, a single, fixed‐dose combination tablet comprising sumatriptan 85 mg and naproxen sodium 500 mg, was developed with the rationale of targeting multiple putative mechanisms involved in the pathogenesis of migraine to optimise acute migraine care.

**Methods:**

A narrative review of clinical trials investigating sumatriptan‐naproxen sodium for both adults and adolescents was performed in March 2024.

**Results:**

Across a total of 14 clinical trials in nine publications, sumatriptan‐naproxen sodium offered greater efficacy for 2‐h pain freedom (14/14) and sustained pain‐free response up to 24 h (13/14) compared with monotherapy and/or placebo for both adult and adolescent study participants with an acceptable and well‐tolerated adverse effect profile. Clinical trial data also demonstrates the effectiveness of sumatriptan‐naproxen sodium in participants with allodynia, probable migraine, menstrual‐related migraine and those with poor responses to acute, non‐specific, migraine medication.

**Conclusions:**

Multi‐mechanistic therapeutic agents offer an opportunity to optimise acute medications by targeting multiple mediators involved in the pathogenesis of migraine. Sumatriptan‐naproxen sodium resulted in greater initial and sustained pain freedom, compared with either sumatriptan, naproxen‐sodium and/or placebo, for the treatment of single or multiple attacks of migraine across both adult and adolescent study populations.

## INTRODUCTION

Migraine is a highly prevalent [1], disabling neurological disease that is marked by attacks of moderate to severe head pain, usually throbbing in quality that, when left untreated, typically lasts between 4 to 72 h. It is associated with photophobia, phonophobia, nausea and/or vomiting [2]. Varied responses to acute medications used for migraine have been observed, with over one‐third (34.5%) of patients reporting insufficient headache relief [3]. Migraine has a complex pathophysiology, known only in part, that involves multiple componenets [4, 5]. In this context, multi‐mechanistic therapeutic agents have been developed as optimal acute treatment approaches with the physiological rationale of targeting multiple putative mechanisms involved in the pathogenesis of the disorder [6].

Sumatriptan‐naproxen sodium, a single, fixed‐dose combination tablet comprising sumatriptan 85 mg and naproxen sodium 500 mg (hereafter denoted sumatriptan‐naproxen sodium), was approved by the US Food and Drug Administration in April 2008 and is now approved in several European countries, as a prescribed medication [6]. It is formulated as a bilayer tablet that includes RT technology (RT: fast disintegrating/rapid release tablet), enabling rapid disintegration and release of sumatriptan, thereby promoting content dispersal [7]. Triptans and nonsteroidal anti‐inflammatory drugs (NSAIDs) may target separate aspects of the potential pathophysiology of migraine. Together, hypothetically, they prevent or reduce both peripheral activation of central pain pathways, and central pathway activation, during the early stages of a migraine attack [8, 9] and the later‐developing central sensitization that is independent of peripheral input [10].

The superior effectiveness of the sumatriptan‐naproxen combination tablet over placebo and its constituent elements have been reported in multiple, replicate, randomized, placebo‐controlled studies of single attacks [6, 11] and multiple attacks of migraine [12, 13] across varying intensities of pain. The data suggest the medications work in synergy to produce more effective acute relief of migraine symptoms [14]. In addition, other clinical benefits, such as increased patient satisfaction, improved functioning and improved migraine‐specific quality of life indicators, have been reported [15, 16, 17]. Notably, participants in the studies were without known sumatriptan contraindications [18].

Herein, we present the results of a narrative review addressing the pharmacodynamics, pharmacokinetics, therapeutic efficacy and tolerability of sumatriptan‐naproxen sodium across a range of populations with migraine, including adults and adolescents, alongside individuals with poor responses to short‐acting triptans, menstrual migraine, probable migraine and allodynia. We consider crossover trial data comparing the effectiveness of sumatriptan‐naproxen sodium to its key counterparts in the acute management of migraine and acknowledge the missing data that must be collected to place this medication correctly into the treatment armamentarium of migraine.

## PHARMACODYNAMIC PROPERTIES

Sumatriptan is a highly selective 5‐hydroxytryptamine (5‐HT) receptor agonist that exerts its effects primarily through the 5HT_1B/1D_ receptors, with much lesser effects on the 5‐HT_1A_, 5‐HT_1E_ or 5‐HT_1F_ receptors [4, 19]. Vascular 5HT_1B_ receptors are mainly located in the cerebral and dural vessels, whilst 5HT_1D_ receptors are located in nervous tissue [19]. Activation of these receptors results in the vasoconstriction of large cerebral and meningeal blood vessels, reduction of neurogenic vasodilation [20, 21] and decreased transmission of pain impulses from second‐order neurons to the trigeminal nucleus caudalis [8]. Triptans may impair the activation of central pathways during the early stages of a migraine attack by inhibiting transmission between peripheral and central neurons [22].

Naproxen, a NSAID, inhibits the biosynthesis of prostanoids via competitively binding to inhibit both cyclooxygenase (COX) isoenzymes, COX‐1 and COX‐2 [23, 24]. It has greater COX‐1 selectivity, providing a favourable cardiovascular safety profile amongst NSAIDs [23, 24], resulting in effective analgesic and anti‐inflammatory effects [23]. COX‐1 and COX‐2 catalyse the conversion of free arachidonic acid to prostaglandin G_2_ (PGG_2_) and then to PGH_2_ [25]. Tissue‐specific isomerases and synthases further transform PGH_2_ into various prostanoids, such as prostaglandin (PG)E_2_, prostacyclin (PGI_2_), PGD_2_, PGF_2α_ and thromboxane (Tx)A_2_ [25]. Through this mechanism, some suggest, based on laboratory data, that NSAIDs may reduce meningeal inflammation, which may contribute to pain and neuronal activation [26], or by direct effects on second‐order trigeminocervical neurons [27].

When combined, sumatriptan and naproxen sodium may target different aspects of the putative pathophysiology of migraine, such that in combination they may provide a more marked positive effect in the acute treatment of migraine, possibly by reducing or preventing both the initial peripheral activation of central pathways during the early stages of a migraine attack and the subsequent development of central sensitization, which occurs independently of peripheral input [10].

## PHARMACOKINETIC PROPERTIES OF SUMATRIPTAN‐NAPROXEN SODIUM

The unique pharmacokinetic and safety profiles of sumatriptan‐naproxen sodium distinct from that of sumatriptan and naproxen have been described in a total of six open‐label studies, all of which are reported in a single publication by Haberer and colleagues [28] (Table 1).

**TABLE 1:** Summary of the pharmacokinetic profiles for comparable doses of the sumatriptan 85 mg‐naproxen sodium 500 mg combination tablet (abbreviated CT sumatriptan‐naproxen), naproxen sodium 500 mg, sumatriptan 85 mg (non‐RT) and sumatriptan 85 mg (RT) and naproxen sodium 500 mg administered as two separate tablets (abbreviated sumatriptan+naproxen).

	Naproxen pharmacokinetic parameters				Sumatriptan pharmacokinetic parameters			
	Study 1		Study 2		Study 1		Study 2	
	CT sumatriptan‐naproxen	Naproxen sodium 500 mg	CT sumatriptan‐naproxen	Sumatriptan + naproxen	CT sumatriptan‐naproxen	Sumatriptan 85 mg (non‐RT)	CT sumatriptan‐naproxen	Sumatriptan + naproxen
Subjects (*n*)	16	16	14	14	16	16	14	14
AUC 0–∞ (h * μg/mL) geometric mean (95% CI)	1512 (1407, 1625)	1449 (1301, 1613)	1234 (1085, 1405)	1214 (1085, 1358)	266 (231.7, 304.6)	241 (205.6, 281.5)	252 (203, 313)	246 (206, 295)
*C*_max_ (μg/mL) geometric mean (95% CI)	69.9 (61.7, 79.1)	95.4 (87.2–104.5)	56.9 (50.8, 63.7)	60.9 (55.4, 66.9)	69.6 (56.6, 85.6)	53.1 (44.9, 62.7)	52.8 (42.8, 65.0)	61.2 (49.0, 76.4)
*T*_max_ (h) median value (min‐max)	6.0 (0.5, 8.0)	1.0 (0.7–3.0)	6.0 (0.5, 6.0)	4.0 (0.5, 9.0)	1.0 (0.5, 2.0)	1.2 (0.5, 4.0)	1.8 (0.5, 4.0)	1.5 (0.5, 4.0)

*Note*: Adapted from Haberer and colleagues [28].

Abbreviations: AUC, area under the curve; CI, confidence interval; *C*_max_, peak concentration; RT, fast disintegrating/rapid release tablet; *T*_max_, median time to maximum concentration.

The most prominent finding was the consistent delay observed in naproxen absorption when administered in combination with sumatriptan 85 mg, with an average peak concentration (*C*_max_) approximately 27%–35% lower and a median time to maximum concentration (*t*_max_) averaging 6 h (5–8 h) compared with monotherapy of naproxen sodium at doses of 500 mg: *t*_max:_ 1 (0.7–3.0) h. Despite the slower absorption of naproxen, the overall systemic exposure (area under the curve, AUC) of naproxen, when administered as a combination tablet, was comparable to exposure from a single naproxen tablet. This suggests that naproxen may contribute to the sustained efficacy of sumatriptan‐naproxen sodium in keeping with its delayed *t*_max_ and observed long half‐life of 12–17 h [23, 28]. The absorption and exposure (AUC and *C*_max_) of sumatriptan 85 mg delivered from the combination tablet was equally similar to that of the commercially available sumatriptan 100 mg (RT) tablet [28]. Exposure of sumatriptan from the combination tablet was approximately 15% greater than that expected from a single sumatriptan 85 mg tablet, whilst the median sumatriptan *t*_max_ occurred 30 min earlier than monotherapy using sumatriptan alone, suggesting a slightly quicker absorption rate of the combination tablet [28] (Table 1).

Furthermore, a second dose of sumatriptan‐naproxen sodium, taken 2 h after the initial dose, was shown to be safe with minimal alterations of the pharmacokinetic profile and without an increased incidence of adverse events compared with that of a single dose, suggesting that the medication can be taken safely in patients with partially resolved migraine attacks [28]. In addition, the administration of subcutaneous sumatriptan 4 and 6 mg administered 2 h after a single dose of sumatriptan‐naproxen sodium demonstrated that sumatriptan exposure did not exceed that of two sumatriptan 100 mg tablets [29].

No differences in the bioavailability and *t*_max_ of sumatriptan‐naproxen sodium were seen between healthy control subjects and adult migraineurs. Similarly, the administration of sumatriptan‐naproxen sodium with food did not affect the bioavailability; however, the median time to maximal concentration of sumatriptan was found to be delayed by approximately 40 min, whilst no differences were observed for the *t*_max_ of naproxen.

## THERAPEUTIC EFFICACY OF SUMATRIPTAN‐NAPROXEN SODIUM

Sumatriptan‐naproxen sodium has been studied in randomized, placebo‐controlled clinical trials with positive results in the acute treatment of migraine for both the adult and adolescent populations compared with placebo and its individual constituents, as shown in Table 2. A summary of the efficacy and safety data of sumatriptan‐naproxen can be seen in Table 3.

**TABLE 2:** Synopsis of the regulatory clinical trials for the treatment with the fixed combination sumatriptan 85 mg (RT technology)/naproxen sodium 500 mg.

Trial	Trials (*n*)	Comparator arms	Single or multiple migraine attack	Early or late intervention
Adult				
*Pivotal studies* (Brandes JL et al. JAMA 2007;297:1443–1454) [6]	2	Sumatriptan 85 mg Naproxen 500 mg Placebo	Single	Late (moderate/severe pain)
*Early intervention studies* (Silberstein S et al. Neurology 2008;71:114–121) [11]	2	Placebo	Single	Early (mild pain, within 1 h)
*Consistency of response studies* (Lipton R et al. Cephalalgia 2009;29:826–836) [12]	2	Placebo	Multiple	Early (mild pain, within 1 h)
*Randomized controlled trial* (Calhoun and Ford. Postgrad Med 2014;126(2):86–90) [13]	1	Placebo	Multiple	Early (mild head or neck pain, within 30 min)
*Triptan poor response studies* (Mathew NT et al. Headache 2009;49(7):971–982) [26]	2	Placebo	Single	Early (mild pain, within 1 h)
*Comparative study* (Landy S et al. Ther Adv Neurol Disord 2013 Sep;6(5):279–286) [58]	1	Sumatriptan 100 mg and naproxen sodium 440 mg administered concomitantly	Multiple	Unclear
*Comparative study* (Derosier F et al. Headache2. 2012 Apr;52(4):530–543) [32]	1	Butalbital medication (BCM—50 mg butalbital, 325 mg acetaminophen, 40 mg caffeine) Placebo	Multiple	Late (moderate/severe pain)
*Menstrual‐related migraine studies* (Mannix LK et al. Obstet Gynecol 2009;114:106–113) [48]	2	Placebo	Single	Early (mild pain, within 1 h)
*Probable migraine without aura studies* (Silberstein S et al. Cephalalgia 2014 Apr;34(4):268–279) [50]	1	Placebo	Single	Late (moderate/severe pain)
*Allodynia in migraine studies* (Landy S et al. Headache 2012 Jan;52(1):133–1339) [52]	1	None	Multiple	Early (mild pain, within 30 min)
*One‐year single‐arm safety study* (Winner P et al. Mayo Clin Proc 2007;82:61–68) [55]	1	None	Multiple	Late (moderate/severe pain)
Adolescent (12–17 years)				
*Randomized controlled trial* (Derosier F et al. Pediatrics 2012;129(6):e1411‐e1420) [38]	1	Sumatriptan‐naproxen sodium: 10/60 mg, 30/180 mg, 85/500 mg Placebo	Single	Late (moderate/severe pain)
*Consistency of response studies* (Winner P et al. Headache 2015 Apr;55(4):519–528) [39]	1	Placebo	Multiple	Early (mild pain, within 1 h)
*One‐year single‐arm safety study* (McDonald SA et al. Headache 2011 Oct;51(9):1374–1387) [57]	1	None	Multiple	Early (mild pain, within 1 h)

*Note*: References [6, 11, 12, 13, 26, 33, 35, 42, 43, 56].

Abbreviation: RT, fast disintegrating/rapid release tablet.

**TABLE 3:** Pooled summary of efficacy and safety data for sumatriptan 50 mg or 85 mg plus naproxen 500 mg compared with placebo for migraine.

Outcome	Probable outcome with comparator	Probable outcome with intervention	NNT or NNH (95% CI)	Studies, attacks, events	Quality of the evidence (GRADE)	Comments
Efficacy findings						
Pain‐free at 2 h for moderate to severe baseline pain	77 in 1000	280 in 1000	RR 3.7 (2.8–4.5) NNT 4.9 (4.3–5.7)	4 studies, 2596 attacks, 462 events	High	Adequate numbers of studies and attacks, study quality good, consistency of response
Pain‐free at 2 h for mild baseline pain	180 in 1000	500 in 1000	RR 2.8 (2.4–3.1) NNT 3.1 (2.9–3.5)	8 studies, 3395 attacks, 1252 events	High	Adequate numbers of studies and attacks, study quality good, consistency of response
Headache relief at 2 h for moderate to severe baseline pain	270 in 1000	580 in 1000	RR 2.2 (2.0–2.4) NNT 3.2 (2.9–3.6)	4 studies, 2596 attacks, 1107 events	High	Adequate numbers of studies and attacks, study quality good, consistency of response
Sustained pain‐free during the 24‐h post‐dose for moderate to severe baseline pain	60 in 1000	200 in 1000	RR 3.4 (2.7–4.4) NNT 7.9 (5.9–8.5)	4 studies, 2596 attacks, 339 events	Moderate	Adequate numbers of studies and attacks, study quality good, consistency of response. Downgraded because of threat from potential publication bias with modest effect size and modest number of events
Sustained pain‐free during the 24‐h post‐dose for mild baseline pain	120 in 1000	370 in 1000	RR 3.0 (2.6–3.6) NNT 4.1 (3.7–4.6)	8 studies, 3396 attacks, 907 events	High	Adequate numbers of studies and attacks, study quality good, consistency of response
Sustained headache relief during the 24‐h post‐dose for moderate or severe baseline pain	160 in 1000	430 in 1000	RR 2.6 (2.3–3.0) NNT 3.8 (3.4–4.3)	4 studies, 2596 attacks, 768 events	High	Adequate numbers of studies and attacks, study quality good, consistency of response
Safety and tolerability findings						
At least 1 AE during treatment for moderate to severe baseline pain	120 in 1000	210 in 1000	RR 2.0 (1.6–2.4) NNH 11 (8.3–15)	4 studies, 2793 attacks, 465 events	Moderate	Adequate numbers of studies and attacks, study quality good, consistency of response. Downgraded because of threat from potential publication bias with modest effect size
At least 1 AE during treatment for mild baseline pain	82 in 1000	140 in 1000	RR 1.5 (1.2–1.9) NNH 18 (13–30)	6 studies, 2823 attacks, 329 events	Moderate	Adequate numbers of studies and attacks, study quality good, consistency of response. Downgraded because of threat from potential publication bias with modest effect size and modest number of events
Serious AE (all levels of baseline pain)	No events	1 event possibly related to intervention				

*Note*: With permission from Law and colleagues [30].

Abbreviations: AE, adverse event; GRADE, Grading of Recommendations, Assessment, Development, and Evaluations; NNH, number needed to harm; NNT, number needed to treat; RR, relative risk.

## ADULT MIGRAINE POPULATION

### Single attack of migraine

Sumatriptan‐naproxen sodium was first investigated against placebo and its individual components, sumatriptan 85 mg and naproxen sodium 500 mg, for the treatment of a single migraine attack with moderate‐to‐severe pain in 2007 using two replicate, randomized, parallel‐group studies [6], shown in Tables 4 and 5. Of those enrolled, participants predominantly had a diagnosis of migraine without aura (71%–79%), most were female (84%–89%) and White (86%–90%). The mean age of participants across the two studies was 39.4 ± 11.2 and 40.3 ± 11.4 years, respectively. Participants had at least a 6‐month history of migraine with or without aura and had a range of 2–6 moderate or severe migraine episodes in the 3 months preceding the screening visit. Notably, participants were eligible for the studies regardless of whether they were triptan‐naïve. Associated symptoms of migraine included photophobia (79%–83%), phonophobia (74%–83%), movement sensitivity (86%–90%) and nausea (41%–56%) across both studies and treatment groups.

**TABLE 4:** Outcomes of 2‐h pain‐free responses and sustained pain‐free responses through to 24 h after treatment with sumatriptan 85 mg‐naproxen sodium 500 mg combination tablet (abbreviated sumatriptan‐naproxen sodium).

Trial	Studies (*n*)	Participants, *n* (%)				Sumatriptan‐naproxen sodium vs. placebo *p*‐value	Sumatriptan‐naproxen sodium vs. sumatriptan *p*‐value
		Sumatriptan‐naproxen sodium	Placebo	Sumatriptan	Naproxen‐sodium		
Outcome: Pain‐free at 2 h							
(Brandes JL et al. JAMA 2007;297:1443–1454) [6]	Study 1 Study 2	125 (34**) 107 (30**)	33 (9) 37 (10)	90 (25) 82 (23)	53 (15) 57 (16)	<0.001 <0.001	0.009 0.02
(Silberstein S et al. Neurology 2008;71(2):114–121) [11]	Study 1 Study 2	146 (52**) 141 (51**)	50 (17) 39 (15)			<0.001 <0.001	
(Calhoun AH and Ford S. Postgrad Med 2014;126(2):86–90) [13]	Study 1	28 (64**)	14 (33)			<0.001	
(Mathew NT et al. Headache 2009;49(7):971–982) [26]	Study 1 Study 2	54 (40**) 59 (44**)	23 (17) 19 (14)			<0.001 <0.001	
(Derosier F et al. Pediatrics 2012;129(6):e1411‐e1420) [38]	Study 1	36 (24*)	14 (10)			0.003	
(Mannix LK et al. Obstet Gynecol 2009;114(1):106–113) [48]	Study 1 Study 2	63 (42**) 79 (52**)	37 (23) 35 (22)			<0.001 <0.001	
(Silberstein S et al. Cephalalgia 2014;34(4):268–279) [50]	Study 1	64 (29**)	24 (11)			<0.001	
Outcome: Sustained pain‐free response (2–24 h)							
(Brandes JL et al. JAMA 2007;297:1443–1454) [6]	Study 1 Study 2	90 (25**) 83 (23**)	30 (8) 25 (7)	59 (16) 51 (14)	37 (10) 37 (10)	<0.001 <0.001	0.009 <0.001
(Silberstein S et al. Neurology 2008;71(2):114–121) [11]	Study 1 Study 2	126 (45**) 110 (40**)	35 (12) 36 (14)			<0.001 <0.001	
(Calhoun AH and Ford S. Postgrad Med 2014;126(2):86–90) [13]	Study 1	30 (69**)	10 (23)			<0.001	
(Mathew NT et al. Headache 2009;49(7):971–982) [26]	Study 1 Study 2	35 (26**) 42 (31**)	11 (8) 11 (8)			<0.001 <0.001	
(Derosier F et al. Pediatrics 2012;129(6):e1411‐e1420.) [38]	Study 1	35 (23*)	13 (9)			0.002	
(Mannix LK et al. Obstet Gynecol 2009;114(1):106–113) [48]	Study 1 Study 2	44 (29*) 57 (38**)	29 (18) 16 (10)			<0.05 <0.001	
(Silberstein S et al. Cephalalgia 2014;34(4):268–279) [50]	Study 1	53 (24**)	20 (9)			<0.001	

*Note*: References [6, 11, 13, 26, 42, 56]. Note that data refer to the number of participants (%). Trials that reported data in respect of migraine attacks were not included in the table [12, 39, 52]. *P‐*values for active treatment versus placebo or sumatriptan.

* *p* < 0.05, unless specified.

** *p* < 0.001.

**TABLE 5:** Other common efficacy outcomes for sumatriptan 85 mg‐naproxen sodium 500 mg combination tablet (abbreviated sumatriptan‐naproxen sodium).

Trial	Studies (*n*)	Participants, *n* (%)				Sumatriptan‐naproxen sodium vs. placebo *p*‐value	Sumatriptan‐naproxen sodium vs. sumatriptan *p*‐value
		Sumatriptan‐naproxen sodium	Placebo	Sumatriptan	Naproxen‐sodium		
Outcome: Migraine‐free at 2 h							
(Silberstein S et al. Neurology 2008;71(2):114–121) [11]	Study 1 Study 2	126 (45) 127 (46)	44 (15) 36 (14)			NR	
(Mathew NT et al. Headache 2009;49(7):971–982) [26]	Study 1 Study 2	47 (35**) 46 (35**)	19 (14) 15 (11)			<0.001 <0.001	
Outcome: Use of rescue medication through 24 h post‐dose							
(Brandes JL et al. JAMA 2007;297:1443–1454) [6]	Study 1 Study 2	81 (22**) 83 (23**)	192 (53) 223 (58)	115 (32) 137 (38)	135 (38) 143 (39)	<0.001 <0.001	0.004 <0.001
(Silberstein S et al. Neurology 2008;71(2):114–121) [11]	Study 1 Study 2	56 (20**) 47 (16**)	130 (47) 117 (45)			<0.001 <0.001	
(Mathew NT et al. Headache 2009;49(7):971–982) [26]	Study 1 Study 2	39 (29**) 29 (22**)	84 (63) 73 (55)			<0.001 <0.001	
(Derosier F et al. Pediatrics 2012;129(6):e1411‐e1420) [38]	Study 1	21 (14**)	47 (32)			<0.001	
(Silberstein S et al. Cephalalgia 2014;34(4):268–279) [50]	Study 1	61 (27**)	101 (46)			<0.001	
Outcome: Absence of photophobia at 2 h							
(Brandes JL et al. JAMA 2007;297:1443–1454) [6]	Study 1 Study 2	211 (58) 180 (50)	131 (36) 122 (32)	173 (48) 166 (46)	166 (47) 148 (41)	<0.001 <0.001	0.007 0.22
(Derosier F et al. Pediatrics 2012;129(6):e1411‐e1420) [38]	Study 1	89 (59*)	59 (41)			0.002	
Outcome: Presence of photophobia at 2 h							
(Silberstein S et al. Neurology 2008;71(2):114–121) [11]	Study 1 Study 2	85 (31**) 59 (22**)	165 (57) 141 (55)			<0.001 <0.001	
(Mathew NT et al. Headache 2009;49(7):971–982) [26]	Study 1 Study 2	57 (42**) 48 (36**)	86 (65) 84 (65)			<0.001 <0.001	
(Silberstein S et al. Cephalalgia 2014;34(4):268–279) [50]	Study 1	46 (21)	60 (27)			NS	
Outcome: Absence of photophobia at 4 h							
(Brandes JL et al. JAMA 2007;297:1443–1454) [6]	Study 1 Study 2	271 (74) 248 (69)	137 (38) 144 (38)	221 (61) 213 (59)	202 (57) 185 (51)	<0.001 <0.001	<0.001 0.004
Outcome: Presence of photophobia at 4 h							
(Silberstein S et al. Neurology 2008;71(2):114–121) [11]	Study 1 Study 2	53 (20**) 32 (12**)	149 (51) 117 (46)			<0.001 <0.001	
(Mathew NT et al. Headache 2009;49(7):971–982) [26]	Study 1 Study 2	33 (24**) 31 (23**)	65 (49) 69 (53)			<0.001 <0.001	
(Silberstein S et al. Cephalalgia 2014;34(4):268–279) [50]	Study 1	60 (27*)	87 (39)			<0.05	
Outcome: Absence of phonophobia at 2 h							
(Brandes JL et al. JAMA 2007;297:1443–1454) [6]	Study 1 Study 2	223 (61**) 204 (56**)	138 (38) 128 (34)	180 (50) 188 (52)	181 (51) 159 (44)	<0.001 <0.001	0.002 0.14
(Derosier F et al. Pediatrics 2012;129(6):e1411–e1420) [38]	Study 1	90 (60*)	60 (42)			0.002	
Outcome: Presence of phonophobia at 2 h							
(Silberstein S et al. Neurology 2008;71(2):114–121) [11]	Study 1 Study 2	71 (26**) 55 (20**)	156 (54) 118 (46)			<0.001 <0.001	
(Mathew NT et al. Headache 2009;49(7):971–982) [26]	Study 1 Study 2	47 (35**) 42 (32**)	73 (55) 68 (52)			<0.001 <0.001	
(Silberstein S et al. Cephalalgia 2014;34(4):268–279) [50]	Study 1	54 (24)	53 (24)			NS	
Outcome: Absence of phonophobia at 4 h							
(Brandes JL et al. JAMA 2007;297:1443–1454) [6]	Study 1 Study 2	274 (75**) 259 (72**)	148 (41) 146 (38)	226 (63) 224 (62)	215 (60) 193 (53)	<0.001 <0.001	<0.001 0.003
Outcome: Presence of phonophobia at 4 h							
(Silberstein S et al. Neurology 2008;71(2):114–121) [11]	Study 1 Study 2	39 (14**) 35 (13**)	130 (45) 113 (44)			<0.001 <0.001	
(Mathew NT et al. Headache 2009;49(7):971–982) [26]	Study 1 Study 2	31 (23**) 27 (20**)	56 (42) 56 (43)			<0.001 <0.001	
(Silberstein S et al. Cephalalgia 2014;34(4):268–279) [50]	Study 1	59 (27*)	83 (38)			<0.05	
Outcome: Absence of nausea at 2 h							
(Brandes JL et al. JAMA 2007;297:1443–1454) [6]	Study 1 Study 2	260 (71) 237 (65)	233 (65) 244 (64)	238 (66) 233 (64)	248 (70) 249 (68)	0.007 0.71	0.07 0.56
(Derosier F et al. Pediatrics 2012;129(6):e1411‐e1420) [38]	Study 1	106 (70)	101 (70)			0.907	
Outcome: Presence of nausea at 2 h							
(Silberstein S et al. Neurology 2008;71(2):114–121) [11]	Study 1 Study 2	46 (17*) 51 (19**)	71 (24) 79 (31)			0.018 <0.001	
(Mathew NT et al. Headache 2009;49(7):971–982) [26]	Study 1 Study 2	39 (29) 33 (25)	45 (34) 43 (33)			NS NS	
(Silberstein S et al. Cephalalgia 2014;34(4):268–279) [50]	Study 1	36 (16)	37 (17)			NS	
Outcome: Absence of nausea at 4 h							
(Brandes JL et al. JAMA 2007;297:1443–1454) [6]	Study 1 Study 2	295 (81**) 266 (73**)	199 (55) 213 (56)	257 (71) 250 (69)	240 (67) 247 (68)	<0.001 <0.001	0.002 0.14
Outcome: Presence of nausea at 4 h							
(Silberstein S et al. Neurology 2008;71(2):114–121) [11]	Study 1 Study 2	29 (11**) 28 (10**)	70 (24) 74 (29)			<0.001 <0.001	
(Mathew NT et al. Headache 2009;49(7):971–982) [26]	Study 1 Study 2	20 (15) 18 (14*)	42 (32) 35 (27)			NS <0.05	
(Silberstein S et al. Cephalalgia 2014;34(4):268–279) [50]	Study 1	51 (23**)	82 (37)			<0.001	

*Note*: References [6, 11, 26, 42]. Note that data refer to the number of participants (%). Trials that reported data in respect of migraine attacks were not included in the table [12, 39, 52]. Migraine‐free defined as no headache pain and no nausea, photophobia or phonophobia. *P‐*values for active treatment versus placebo. Abbreviations: NR, not reported; NS, non‐significant.

* *p* < 0.05, unless specified.

** *p* < 0.001.

**FIGURE 1:**
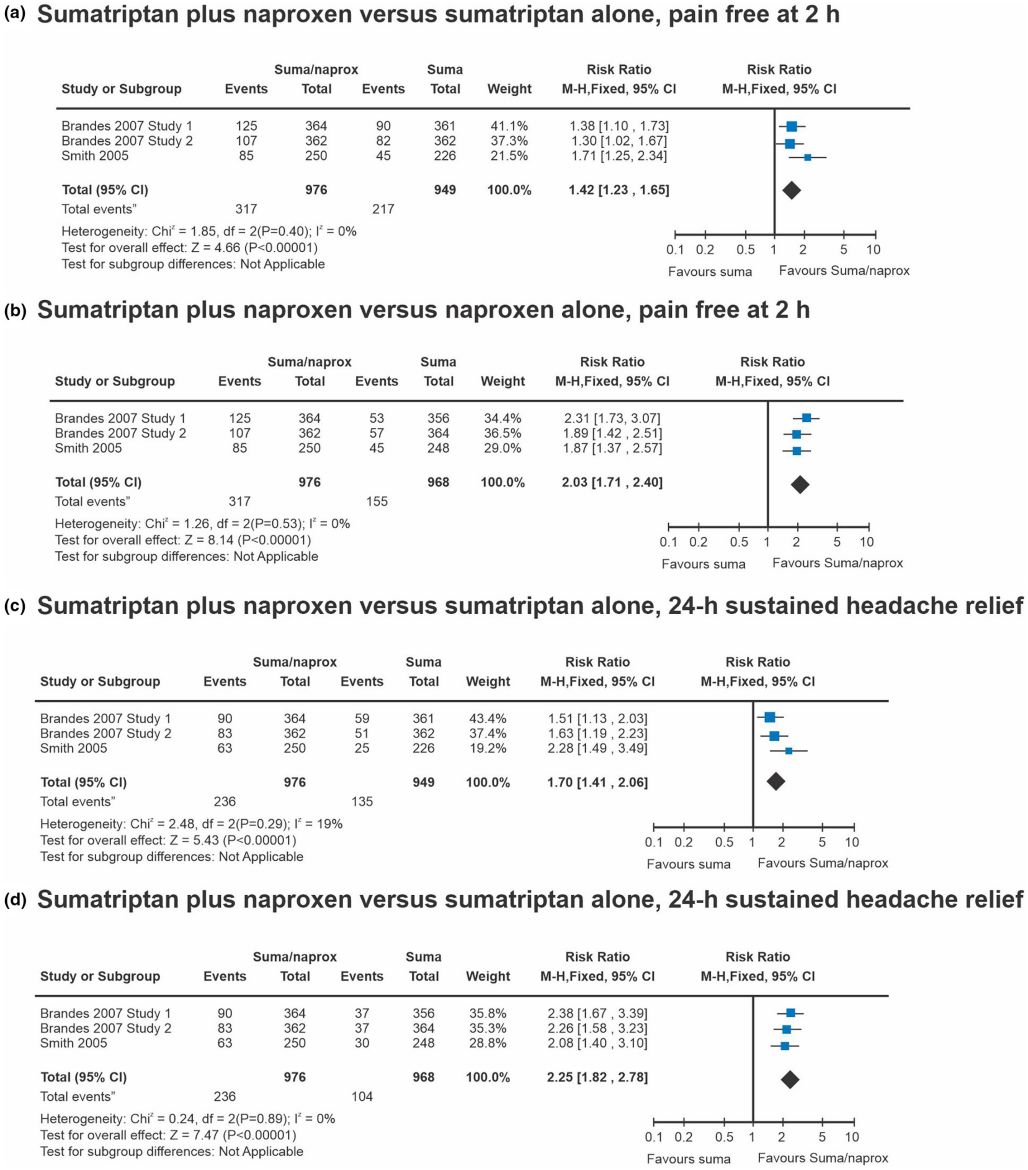
Comparisons of combined sumatriptan‐naproxen sodium versus either drug alone were conducted as part of a Cochrane review published in 2016. All 14 of the separate analyses demonstrated that combined sumatriptan‐naproxen sodium was superior to monotherapy across all efficacy outcomes examined, including in the representative series shown here. The numerical additional effect of combined sumatriptan‐naproxen was larger versus naproxen sodium alone than for sumatriptan alone. (a) Comparison combined sumatriptan‐naproxen sodium versus sumatriptan alone, outcome pain‐free at 2 h; (b) comparison combined sumatriptan‐naproxen sodium versus naproxen alone, outcome pain‐free at 2 h; (c) comparison combined sumatriptan‐naproxen sodium versus sumatriptan alone, outcome 24‐h sustained pain free; and (d) comparison combined sumatriptan‐ naproxen sodium versus naproxen alone, outcome 24‐h sustained pain free. From Law and colleagues [30].

In both studies, sumatriptan–naproxen sodium was superior to placebo and its individual components at the 2‐h post‐dose mark, delivering greater relief from headache [6]. Notably in the first study (*n* = 1461), 65% of participants reported headache relief 2 h post‐dose with sumatriptan‐naproxen sodium, outperforming both sumatriptan monotherapy (55%) and naproxen sodium monotherapy (44%), as well as placebo (28%; *p* < 0.001 for sumatriptan–naproxen sodium, sumatriptan, and naproxen sodium vs. placebo; *p* = 0.009 for sumatriptan–naproxen sodium vs. sumatriptan) [6]. Similar results were seen in the second study (*n* = 1495), with 57% of participants achieving headache relief 2 h post‐dose with sumatriptan‐naproxen sodium, surpassing both sumatriptan monotherapy (50%) and naproxen sodium monotherapy (43%), as well as placebo (29%; *p* < 0.001 for sumatriptan–naproxen sodium, sumatriptan, and naproxen sodium vs. placebo; *p* = 0.03 for sumatriptan–naproxen sodium vs. sumatriptan) [6]. Forest plot of comparison [30] can be seen for the incidence of pain freedom at 2 h in Figure 1, highlighting that sumatriptan‐naproxen sodium produced significantly greater initial pain freedom than its individual constituents of sumatriptan and naproxen sodium alone [6]. Treatment with sumatriptan‐naproxen sodium resulted in a greater occurrence of 24‐h sustained pain freedom amongst 23%–25% of participants compared with placebo (7%–8%; *p* < 0.001 for both studies) and its individual counterparts (sumatriptan monotherapy: 14%–16%: *p* = 0.009 for study 1 and *p* < 0.001 for study 2, naproxen sodium monotherapy: 10%) [6], see Figure 1 [30]; and notably, fewer participants treated with sumatriptan‐naproxen sodium used rescue medication (*p* < 0.001 for both studies for sumatriptan‐naproxen sodium vs. placebo) or experienced headache recurrence (13% for sumatriptan‐naproxen sodium compared with 19%–24%, 16%–22% and 25%–31% for sumatriptan, naproxen sodium and placebo monotherapy) [6]. In study 1, the relief of nausea 2 h post‐dose when treated with sumatriptan‐naproxen sodium was greater than placebo (71% vs. 65%; *p* = 0.007), whilst the alleviation of nausea in the second study was not met (65% vs. 64%; *p* = 0.71), attributed to a baseline discrepancy in the incidence of nausea: present in 56% of subjects in the sumatriptan‐naproxen arm and 49% in the placebo arm. Other efficacy outcome measures can also be seen in Table 5, of which sumatriptan‐naproxen sodium was more effective than placebo in all measures [6].

Silberstein and colleagues [11] additionally investigated an early treatment paradigm using sumatriptan‐naproxen sodium versus placebo to treat a single migraine attack within 1 h of pain onset whilst the pain was still mild. A total of 658 and 647 participants were randomized to treatment across two replicate, randomized, placebo‐controlled trials. Demographics and baseline characteristics were similar amongst the study and treatment groups, with the majority of participants being female (87%–91%), White (84%–88%) and observing a mean age of 39.3 ± 10.6 and 40.8 ± 11.2 years. Of those enrolled, most participants had a diagnosis of migraine without aura (63%–69%). The median time to treatment was 24–30 min for both groups, and 86%–88% of all participants followed an early treatment approach whilst their head pain was mild [11]. Sumatriptan‐naproxen sodium generated more pain‐free responses at 2 h compared with placebo (*p* < 0.001) (Table 4). Pain‐free responses were observed as early as 30 min for both studies (study 1: sumatriptan‐naproxen 5% > 2% placebo, *p* = 0.016; study 2: sumatriptan‐naproxen 6% > 2% placebo, *p* < 0.021) and persisted throughout all time intervals of testing through to 24 h (study 1: sumatriptan‐naproxen 45% > 12% placebo, *p* < 0.001; study 2: sumatriptan‐naproxen 40% > 14% placebo, *p* < 0.001) (Table 4) [11]. The rate of progression from mild to moderate‐to‐severe migraine pain was two to three times higher in participants treated with placebo compared with those treated with sumatriptan‐naproxen sodium from 30 min to 4 h [11]. Importantly, the incidence of traditional migraine‐associated symptoms of nausea, photophobia and phonophobia at 2 and 4 h was significantly lower compared with placebo in both studies (Table 5) [11]. No significant differences were observed in the incidence of vomiting post‐baseline, thought to be secondary to a very low (≤2%) incidence of baseline vomiting amongst participants [11].

### Multiple attacks of migraine

The investigation of sumatriptan‐naproxen sodium's response to multiple migraine attacks was prompted by initial data collected by Smith and colleagues [17] who evaluated participant‐reported pain responses, satisfaction with treatment and health‐related quality of life (HRQOL) across a total of 24,485 migraine attacks over a 12‐month period in an open‐label study. Of the 600 participants enrolled, nearly all (94%, 565/600) participants treated one or more of their migraine attacks with sumatriptan‐naproxen sodium, and 64% self‐selected to remain on the medication across the 12‐month study period. Of the attacks treated, 70% of participants used a single dose of sumatriptan‐naproxen sodium and only 2% of participants required further rescue medication post‐dose. Data collected at the 12‐month interval demonstrated the ongoing therapeutic benefit, with 80% of participants experiencing pain relief and 60% of participants reporting pain freedom at 2 h following a single dose of sumatriptan‐naproxen sodium, suggesting that the response to sumatriptan‐naproxen sodium was maintained across multiple attacks of moderate‐to‐severe migraine. Migraine‐specific HRQOL using the Migraine‐Specific Quality of Life Questionnaire (MSQ) showed that over half (56%–65%) of participants experienced at least a minimal clinically important improvement throughout the 12‐month period whilst using sumatriptan‐naproxen sodium. Satisfaction ratings doubled for several items in the Patient Perception of Migraine Questionnaire (PPMQ), including speed and duration of relief, return to activity and overall treatment effects. These scores persisted throughout the 12 months, highlighting sustained improvements in migraine‐related quality of life compared with conventional therapy, whilst satisfaction with sumatriptan‐naproxen sodium's ability to relieve pain was 90% and 86% at month 3 and 12, respectively, compared with a 52% rating for previous treatment [17].

To investigate further, Lipton and colleagues evaluated the consistency of response to sumatriptan‐naproxen sodium in adults with multiple attacks of migraine using two identical, randomized, placebo‐controlled crossover studies [12]. In study 1, 570 participants treated 1693 attacks with sumatriptan‐naproxen sodium and 424 attacks with placebo, whilst in study 2, 565 participants treated 1678 attacks with sumatriptan‐naproxen sodium and 422 attacks with placebo. Similar to the aforementioned studies, participants were typically female (89%–90%), White (88%–89%) with a mean number of monthly migraine days ranging from 3.7 ± 1.4 to 3.9 ± 1.5 across both study groups. The study incorporated a crossover design whereby participants were given random insertions of interspersed placebo throughout the study, with the aim to facilitate more stable estimates in response to active treatment whilst eliminating common drawbacks of other study designs, namely, participation bias from uncontrolled, open‐label studies as well as rates of high attrition and attack‐to‐attack carryover effects in multiple attack, placebo‐controlled study designs [31]. Participants were asked to practise early intervention by treating migraine attacks within 1 h of pain onset when the pain was mild.

Compared with placebo, sumatriptan‐naproxen sodium conferred higher 2‐h pain‐free response rates (study 1: sumatriptan‐naproxen 52%, placebo 25%; study 2: sumatriptan‐naproxen 50%, placebo 20%; both *p* < 0.001) and 24‐h sustained pain‐free response rates (study 1: sumatriptan‐naproxen 37%, placebo 17%; study 2: sumatriptan‐naproxen 34%, placebo 12%; both *p* < 0.001) [12]. The therapeutic gain, derived from treatment with sumatriptan‐naproxen sodium compared with placebo, was high across both coprimary endpoints (2 h pain free: 28%, 30% and 24 h sustained pain free: 20%, 22% in study 1 and 2, respectively) [12], suggesting that sumatriptan‐naproxen sodium is effective across attacks with no evidence of tolerance to the therapeutic benefits. In both studies, more attacks were characterized as ‘migraine‐free,’ defined as no pain, nausea, vomiting, photophobia or phonophobia and no use of rescue medication, 2 and 4 h post‐dose following treatment with sumatriptan‐naproxen sodium than with placebo (2 h: study 1: sumatriptan‐naproxen 44% > 21% placebo, *p* < 0.001; study 2: sumatriptan‐naproxen 43% > 17% placebo, *p* < 0.001; 4‐h: study 1: sumatriptan‐naproxen 69% > 36% placebo, *p* < 0.001; study 2: sumatriptan‐naproxen 66% > 31% placebo, *p* < 0.001) [12]. In addition to relieving pain, the presence of photophobia, phonophobia and nausea was reduced in participants treated with sumatriptan‐naproxen sodium 2 h post‐dose compared with the placebo group dose (*p* < 0.001 for the presence of photophobia, phonophobia and nausea 2 h post‐dose for both studies, respectively) [12]. Moreover, the use of rescue medication within 24 h of treatment with sumatriptan‐naproxen sodium was reported in fewer patients compared with placebo in both studies (*p* < 0.001 for study 1 and 2, respectively) [12].

Calhoun and Ford [13] performed one further randomized, double‐blinded, placebo‐controlled trial investigating the role of neck pain as a marker of central sensitization in episodic participants treated with sumatriptan‐naproxen sodium utilizing an early treatment approach (*n* = 43). Although focused on the role of neck pain, the study observed a much higher 2‐h pain‐free response in participants treated with sumatriptan‐naproxen sodium compared with placebo (sumatriptan‐naproxen sodium 63.9% vs. 33.3% placebo; *p* < 0.01) and this was sustained through to 24 h (sumatriptan‐naproxen 69.1% vs. 23.3% placebo; *p* < 0.01) [13].

### Comparisons with other migraine therapeutics

Limited data exist on the comparisons of sumatriptan‐naproxen sodium with other acute migraine therapies and when used in conjunction with conventional migraine preventives and calcitonin gene‐related peptide (CGRP) monoclonal antibody therapies. Head‐to‐head trials have compared sumatriptan‐naproxen sodium to butalbital/acetaminophen/caffeine and short‐acting triptans, the two most commonly prescribed acute medications in the United States and Europe [26, 32]. However, no trials have compared sumatriptan‐naproxen sodium to other acute medications, such as simple analgesics, individual triptans, gepants and other combination treatments like frovatriptan‐dexketoprofen. Moreover, no trials have evaluated the concurrent use of sumatriptan‐naproxen sodium with the parallel use of classic and CGRP‐targeted prophylactics, highlighting the need for additional data to properly position this medication within the migraine treatment paradigm.

In the United States, sumatriptan‐naproxen sodium has been compared with the most commonly prescribed acute migraine medication, butalbital 50 mg/acetaminophen 325 mg/caffeine 40 mg [32]. Here, a total of 442 participants were enrolled in a phase IIIB, randomized, double‐blind, placebo‐controlled, multiple‐attack crossover study where participants treated three migraine attacks with either placebo, sumatriptan‐naproxen sodium or a butalbital‐containing combination medication (BCM), comprised of 50 mg butalbital, 325 mg acetaminophen (paracetamol) and 40 mg caffeine. Of the participants enrolled, most (63%) had a diagnosis of migraine without aura, were typically female (88%) and had a mean age of 42.6 (range 18–65) years. The primary endpoint was the percentage of treated attacks with sustained pain‐free response 2–24 h after treatment [32].

No differences were observed in the sustained pain‐free response rates 2–24 h post‐dose between sumatriptan‐naproxen sodium and BCM (*p* = 0.378); however, both treatments demonstrated significantly higher rates of sustained pain freedom when compared with the placebo (sumatriptan‐naproxen: *p* = 0.011, BCM: *p* = 0.047) [32]. Sumatriptan‐naproxen sodium demonstrated superior efficacy to both BCM and placebo for pain‐free responses observed at 2, 4, 6, 8, 24 and 48 h (*p* < 0.05 vs. both placebo and BCM, respectively) and provided consistent relief of the canonical (i.e., nausea, photophobia and phonophobia) and non‐canonical associated symptoms (i.e., sinus and neck pain) at 4, 6 and 8 h post‐dose (*p* < 0.05), with the single exception of neck pain at 8 h, when compared with BCM [32]. No differences were observed between all treatment groups for the recurrence of head pain [32]. A total of 23% of study participants reported at least one adverse event, with the highest rate (12%) seen in the sumatriptan‐naproxen group compared with that of placebo (10%) and BCM (9%) [32].

Sumatriptan‐naproxen sodium has also emerged as a potential alternative for patients with suboptimal responses to triptan monotherapy, which approximately account for 30% of migraineurs, due to its ability to target more than one mechanism of migraine than monotherapy alone. Through the use of two replicate, randomized, placebo‐controlled, crossover studies, sumatriptan‐naproxen sodium has demonstrated significantly greater effectiveness than placebo in conferring initial, intermediate and sustained freedom from migraine pain and migraine‐associated symptoms of photophobia and phonophobia when administered within 1 h of onset of migraine headache pain [26]. Of the 342 participants randomized into the two studies (study 1: 173, study 2: 169), participants were mostly female (85%–93%), White (88%–92%) and had a mean age of 41.4 ± 10.3 (study 1) and 40.1 ± 11.1 years (study 2). The majority of participants had migraine without aura, with 1–8 migraine attacks monthly and fewer than 15 headache days per month. On average, participants had typically discontinued 3.3 triptans before enrolment, with eletriptan being reported as the most likely to be discontinued followed by sumatriptan. The authors reported that this was likely to be an artefact, owing to the original protocol specification that limited participation only to patients who responded poorly to eletriptan at first. The inclusion criteria were later expanded, because of slow recruitment, to allow patients who had discontinued the use of other short‐acting triptans, namely almotriptan, rizatriptan, sumatriptan or zolmitriptan, because of poor response or intolerance. Frovatriptan and naratriptan were considered to be long‐acting triptans and were not included.

**FIGURE 2:**
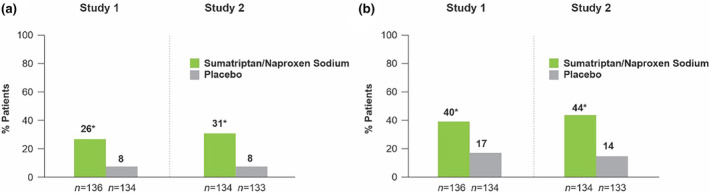
Responses to combination sumatriptan‐naproxen sodium in patients with a history of poor response to triptan monotherapy. (a) Twenty‐four‐hour sustained pain‐free response after dosing with sumatriptan‐naproxen sodium or placebo. (b) Pain‐free response 2 h after dosing with sumatriptan‐naproxen sodium or placebo. From Mathew and colleagues [26].

Sumatriptan‐naproxen sodium was more efficacious than placebo for the percentage of participants with a sustained pain‐free response (2–24 h) in both studies (study 1: sumatriptan‐naproxen 26 > 8% placebo, study 2: sumatriptan‐naproxen 31 > 8% placebo, *p* < 0.001 for both studies) (Figure 2) [26]. Moreover, sumatriptan‐naproxen sodium generated greater pain‐free responses at the 2‐h mark post‐dose administration (study 1: sumatriptan‐naproxen 40 > 17% placebo, study 2: sumatriptan‐naproxen 44 > 14% placebo, *p* < 0.001, respectively) (Figure 2) [26]. No period effect was observed. Traditionally associated symptoms of migraine, such as photophobia and phonophobia, were further reduced at 2, 4, 8, and 2 through 24 h following treatment with sumatriptan‐naproxen sodium compared with placebo (*p* < 0.05 for phonophobia 8 h post‐dose; *p* < 0.001 for all other measures and time points) [26]. Whilst for the incidence of nausea, sumatriptan‐naproxen sodium was significantly more effective than placebo 8 h post‐dose and 2 through 24 h post‐dose in study 1 as well as 4, 8, and 2 through 24 h post‐dose in study 2 [26]. A full breakdown of the other efficacy endpoints is summarized in Table 5 [26].

## ADOLESCENT MIGRAINE POPULATION

The prevalence of migraine amongst children and adolescents ranges from 3% to 10%, depending on specific age groups and country, and increases with age [33]. In the adolescent population, migraine is often characterized by recurrent attacks of bilateral or unilateral, pulsating headache that are typically shorter in duration than those experienced by adults [2]. Migraine therapies commonly used in adolescents, for both acute and preventive treatment, often mirror adult regimes [34]; however, the predominantly favoured acute treatments of the adolescent cohort are ibuprofen and paracetamol, used by 60% of adolescents aged 12–19 years with migraine [35]. Studies evaluating the use of triptans in the adolescent population have suggested efficacy, albeit with an increased risk of minor, non‐serious adverse events; however, clear efficacy for the use of triptans in adolescents remains undecided as a result of the high placebo rates of at least 50% seen in the adolescent population compared with the 35% placebo rate observed in adults [36, 37].

### Single attack of migraine

Derosier and colleagues [38] compared the safety and efficacy of three varying doses of sumatriptan‐naproxen sodium against placebo in the acute treatment of a single, moderate‐to‐severe migraine attack in adolescents aged between 12 and 17 years of age. Of the 490 included participants, the mean age was 14.7 ± 1.72 years; participants were predominantly White (81.0%) and mostly female (58.6%). In the double‐blind phase, participants treated one moderate‐to‐severe migraine with either placebo (*n* = 145) or varying doses of sumatriptan‐naproxen sodium: 10/60 mg (low dose) = 96, 30/180 mg (middle dose) = 97, 85/500 mg (high dose) = 152, all of which were identical in appearance, size, markings, colour and weight.

Sumatriptan‐naproxen sodium demonstrated efficacy compared with placebo for 2‐h pain‐free rates across all three treatment arms: sumatriptan‐naproxen sodium 10/60 mg (29%; adjusted *p* = 0.003), sumatriptan‐naproxen sodium 30/180 mg (27%; adjusted *p* = 0.003) and sumatriptan‐naproxen sodium 85/500 mg (24%; adjusted *p* = 0.003) versus placebo (10%) (Table 4). Post‐hoc primary analyses did not demonstrate significant differences amongst the varying treatment arms or an age‐by‐treatment interaction. Statistically significant differences were observed for the 85/500 mg arm versus placebo for sustained pain‐free 2–24 h (23% vs. 9%; adjusted *p* = 0.008), 2‐h photophobia‐free (59% vs. 41%; adjusted *p* = 0.008) and 2‐h phonophobia‐free (60% vs. 42%; adjusted *p* = 0.008). Aside from 1‐h pain‐free and 2‐h nausea‐free measures, all other secondary endpoints showed a numerical improvement with the 85/500 mg dose compared with the placebo (unadjusted *p* < 0.01) (Table 5) [38].

### Multiple attacks of migraine

Similar results were observed by Winner and colleagues [39] who investigated the use of sumatriptan‐naproxen sodium in a multiple‐attack, crossover study design utilizing an early intervention approach. In the study, 94 adolescents treated a total of 347 attacks, of which 277 attacks were treated with sumatriptan‐naproxen sodium and the remaining 70 with placebo medication. The mean age was 14.7 years, with the majority of participants being female (62%), White (85%) and having a mean number of 4.96 migraine attacks per month.

Across attacks, pain‐free responses at the 2‐h post‐dose mark were greater in the sumatriptan‐naproxen sodium group compared with placebo (sumatriptan‐naproxen 37% vs. 18% placebo; *p* = 0.0038) [39] (Table 4). This was similarly reflected across attacks utilizing an early intervention approach (sumatriptan‐naproxen 32% vs. 18% placebo; *p* = 0.02262) [39]. No differences were observed across attacks for sustained pain freedom (sumatriptan‐naproxen 86% vs. 78% placebo; *p* = 0.1294) [39], with the lower differentiation of sumatriptan‐naproxen sodium from placebo likely attributed to adolescents having, in general, shorter migraine attack durations (Table 4). Similar to that of adults [12], consistent response to sumatriptan‐naproxen sodium was demonstrated across multiple attacks of migraine with approximately half of the participants reporting pain freedom 2 h post‐dose in at least two of the three migraine attacks treated with sumatriptan‐naproxen sodium [39].

## MENSTRUAL MIGRAINE

Menstrual migraine, a common umbrella term that covers the definitions of pure menstrual migraine and menstrually‐related migraine, describes a diagnosis of migraine with or without aura that commonly occurs on or between days −2 to +3 of menstruation in at least two of three consecutive menstrual cycles [2]. Menstrual migraine without aura is estimated to affect up to one‐quarter (18%–25%) of female migraineurs [40, 41, 42, 43], whilst in populations from headache clinics, the proportion of women affected is estimated to be even higher (22%–70%) [44, 45, 46]. In women diagnosed with menstrual migraine, perimenstrual attacks are associated with a significantly longer attack duration, greater work disability, reduced pharmacological response compared with non‐menstrual attacks and the presence of non‐neurological symptoms, mainly dysmenorrhoea, which increase the burden of disease [47]. The management of menstrual migraine is not vastly different to the management of non‐menstrual migraine. However, consideration must be given to the longer attack duration, often requiring several consecutive days of treatment and additional treatment for headache recurrence, and that patients may be relatively refractory to medicines that work in non‐menstrual attacks.

Two replicate, randomized, placebo‐controlled trials investigating sumatriptan‐naproxen sodium showed excellent efficacy in the treatment of a single menstrual migraine attack associated with dysmenorrhoea [48]. The studies included 312 and 311 participants in the intention‐to‐treat (full analysis set), respectively, who were randomly assigned to the study group (*n* = 160 and 151) or the placebo group (*n* = 152 and 160) [48]. Of the participants in study 1, 74% had a diagnosis of menstrual migraine without aura with a median age of onset of 21 years, and similar values were seen in study 2 where 60% of participants had menstrual migraine without aura with a median age of onset of 22 years [48]. Participants had a median of three migraines and five headache days per month, with mean attack duration ranging from 24 h to greater than 72 h for 57%–61% of participants [48].

A greater proportion of participants were observed to be headache‐free 2 h after treatment compared with that of placebo, meeting its primary endpoint (study 1: sumatriptan‐naproxen 42% > 23% placebo, *p* < 0.001; study 2: sumatriptan‐naproxen 52% > 22% placebo, *p* < 0.001) [48] (Table 4). Notably, patients reported pain freedom as early as 1‐h post‐dose in study 2 (sumatriptan‐naproxen 29% > 8% placebo, *p* < 0.001) [48]. The percentage of patients reporting pain freedom was roughly twice that in the sumatriptan‐naproxen sodium group compared with the placebo group at the 4‐h interval mark for both studies, respectively (study 1: sumatriptan‐naproxen 60% > 36% placebo, *p* < 0.001; study 2: sumatriptan‐naproxen 66% > 30% placebo, *p* < 0.001) [48]. Similarly, the rate of sustained pain‐free responses up to 24 h post‐dose was higher amongst participants treated with sumatriptan‐naproxen sodium than those given the placebo (study 1: sumatriptan‐naproxen 29% > 18% placebo, *p* < 0.05; study 2: sumatriptan‐naproxen 38% > 10% placebo, *p* < 0.001) [48]. This was additionally sustained through to 48 h (study 1: sumatriptan‐naproxen 26% > 17% placebo, *p* < 0.05; study 2: sumatriptan‐naproxen 28% > 8% placebo, *p* < 0.001) [48]. In both studies, sumatriptan‐naproxen sodium was statistically superior to placebo (*p* < 0.05) and reduced the requirement for rescue medication, for both headache and menstrual symptoms [48]. Statistically significant differences between the study and placebo group favoured the use of sumatriptan‐naproxen to help relieve non‐painful menstrual symptoms such as bloating, fatigue and irritability; however, no significant differences between the groups were observed for menstrual pain symptoms such as overall pain, abdominal pain and back pain [48]. It was acknowledged that baseline menstrual pain data were not collected, and pain intensity was only measured for 4 h. In addition, the authors highlight that the unique pharmacokinetic profile of sumatriptan‐naproxen sodium resulting in a delayed and blunted maximal plasma concentration may have affected the therapeutic window [48]. Further post‐hoc analysis revealed that sumatriptan‐naproxen sodium worked better in individuals with no or mild menstrual symptoms at baseline (2‐h pain‐free response: 61%, 69%) compared with others with at least one moderate to severe baseline menstrual symptom (2‐h pain‐free response: 32%, 42%) [48], suggesting that individuals with comorbid menstrual migraine and moderate‐to‐severe menstrual symptoms may show enhanced pain perception compared with those with more mild symptoms [49].

## PROBABLE MIGRAINE WITHOUT AURA

Probable migraine is defined by the International Headache Classification of Disorders, Third Edition (ICHD‐3) as a headache that meets all except one of the diagnostic criteria for migraine with or without aura [2]. It has an estimated prevalence of 3%–10%; however, it is likely underrecognized, with a majority of patients being misdiagnosed as having sinus or tension‐type headache [50]. Standard‐of‐care treatment approach for probable migraine resembles that of migraine based on the assumption that the pathophysiology and treatment response profiles are similar [50]. Only one randomized, double‐blinded, placebo‐controlled trial has been performed investigating the use of sumatriptan‐naproxen sodium in those with probable migraine [50]. In a population of 443 randomized adult participants (*n* = 222 sumatriptan‐naproxen sodium, *n* = 221 placebo) where the typical participant was female (72%–77%), White (81%) with a mean age of 35.1 ± 11.57 years in the study group and 35.8 ± 10.91 years in the placebo group, sumatriptan‐naproxen sodium demonstrated greater values for 2‐h pain freedom (sumatriptan‐naproxen 29% vs. 11% placebo, *p* < 0.001) and sustained pain‐free responses over 24 h (24% sumatriptan‐naproxen vs. 9% placebo, *p* < 0.001) compared with that of placebo (Table 4) [50]. It further improved “normal” functioning at both 2 h (*p* = 0.036) and 4 h post‐dose (*p* < 0.001) compared with placebo; however, no differences were seen in productivity between the two groups [50]. A greater proportion of participants reported better effectiveness and overall treatment satisfaction compared with placebo or previous therapy, most of which was NSAID therapy (sumatriptan‐naproxen: 62%, placebo: 43%, previous therapy: 29%–31%: *p* < 0.001 vs. placebo and previous medications), whilst 6 of 10 participants were satisfied or very satisfied with the side effects of combination therapy compared with previous therapy (44%) but not compared with placebo (64%) [50].

## ALLODYNIA

Cutaneous allodynia is estimated to affect 63% of migraineurs and is characterized by pain provoked by stimulation of the skin that would ordinarily not produce pain [51]. Sumatriptan‐naproxen sodium has been shown to elicit positive results in the treatment of allodynic patients in an open‐label prospective study, where the dosage was administered within 30 min of symptom onset [52]. Of the 40 participants enrolled, 80% of the cohort had migraine without aura and 95% had an average of two or more positive responses to the Allodynia Questionnaire [52]. Participants were most likely to be female (90%), White (90%), with a mean age of 42.9 ± 8.82 years [52]. The primary endpoint of the study was the percentage of participants who had a sustained pain‐free response (2–24 h) post‐dose and participants' overall satisfaction with sumatriptan–naproxen using the revised Patient Perception of Migraine Questionnaire‐R [52]. Over the 12‐week study period, patients treated four migraine attacks with instructions to initiate treatment within 30 min from the onset of pain whilst the pain was mild. Sustained pain freedom at 24 h was seen in 49% of participants (78/160), whilst 2‐h pain freedom was seen in 59% of participants (94/160) [52]. Across the four migraine attacks, 42.5% of participants were satisfied with treatment [52]. The authors speculate that clinical improvement in allodynic patients may be attributed to a number of factors: the first, that the combination of triptan‐NSAID may disrupt both the peripheral and central sensitization owing to better analgesic relief in these patients; and the second, that early intervention of therapy in those susceptible to allodynia may reduce the progression to central sensitization [52].

## PREGNANCY

No major birth defects were reported in patients exposed to sumatriptan‐naproxen sodium in the first trimester of pregnancy, as reported by the Sumatriptan, Naratriptan, and Treximet Pregnancy Registry [53]. Of the 680 exposed pregnant women which resulted in 689 infants and foetuses across a 16‐year period, the majority (92.1%; 626/680) were exposed to sumatriptan, whilst a smaller proportion was exposed to naratriptan (8.3%; 57/680) and an even smaller proportion to sumatriptan‐naproxen sodium (0.9%; 6/680) [53]. Although the Registry detected no signal of teratogenicity associated with major birth defects for sumatriptan, there is a lack of evidence for its use in pregnancy and the use of sumatriptan‐naproxen sodium must be cautioned in any conclusion, especially in the third trimester due to the risk of foetal abnormalities (patent ductus arteriosus closure and oligohydramnios) [54].

## SAFETY AND TOLERABILITY

Adverse effects reported from the use of sumatriptan‐naproxen sodium include dizziness, paraesthesia, somnolence, nausea, dry mouth and chest discomfort (Table 6). The type and frequency of adverse events reported in the long‐term safety and tolerability studies of sumatriptan‐naproxen sodium are similar to those reported in long‐term studies of sumatriptan monotherapy.

**TABLE 6:** Common adverse effects occurring at a rate of 2% or greater in those taking sumatriptan‐naproxen sodium as reported by two single‐arm tolerability and safety studies conducted over 12 months in the adult and adolescent migraine populations.

Adverse effect	Adult	Adolescent
Sample (*n*)	565	622
At least one adverse event thought to be related to the study drug, *n* (%)	152 (27)	170 (27)
Nausea	34 (6)	55 (9)
Dizziness	17 (3)	25 (4)
Paraesthesia	11 (2)	
Chest discomfort	11 (2)	
Throat tightness	11 (2)	
Dyspepsia	11 (2)	
Upper abdominal pain	11 (2)	
Upper respiratory tract infection		54 (9)
Nasopharyngitis		48 (8)
Sinusitis		37 (6)
Neck pain		24 (4)
Oropharyngeal pain		22 (4)
Worsening of migraine		22 (4)

*Note:* Results adapted from Winner and colleagues [55] and McDonald and colleagues [57].

In a 12‐month, multi‐centre, open‐label study including 600 participants, Winner and colleagues investigated the long‐term safety and tolerability of sumatriptan‐naproxen sodium for the treatment of migraine [55]. Most participants who reported adverse events deemed them mild (17%) or moderate (36%) in severity, with 27% of the overall safety population (*n* = 565) reporting one or more adverse events that were thought to be related to sumatriptan‐naproxen sodium [55]. Adverse events included nausea (6%), muscle tightness (3%), dizziness (3%), dyspepsia (2%) and paraesthesia (2%) [55]. No deaths occurred throughout the study; however, 14 participants (2%) reported one or more serious adverse events with only one, a case of acute coronary syndrome, judged as probably related to treatment [55]. No differences in the incidence of adverse events were observed in those taking two tablets, taken at least 2 h apart, compared with one tablet. A further study concluded that there were no mean changes from baseline blood pressure amongst those taking sumatriptan‐naproxen as compared with sumatriptan or naproxen sodium monotherapy [56].

Similar to the case in adults [55], McDonald and colleagues [57] found that sumatriptan‐naproxen sodium was well‐tolerated in adolescent migraineurs (*n* = 656) over a 12‐month study period (Table 6). There were no new or clinically significant findings of sumatriptan‐naproxen sodium in the safety parameters, as compared with its individual components or to the adverse effect profile in adults [57].

## CONCLUSIONS

The collective data from clinical trials suggest that sumatriptan‐naproxen sodium offers significant improvement in sustained relief and pain‐free responses, presenting an alternative treatment approach for the acute management of migraine in both adult and adolescent populations. Sumatriptan‐naproxen sodium appears to exhibit a synergistic improvement over the individual components of monotherapy alone, offering increased efficacy whilst reducing the need for rescue medication, even in those with previously poor responses to short‐acting triptans. Other cohorts of patients may also benefit from a triptan–NSAID combination, such as those with menstrual migraine, probable migraine and migraine accompanied with allodynia. Moving forward, studies should be undertaken to compare the efficacy, safety and tolerability of sumatriptan‐naproxen sodium with other acute migraine therapeutics, namely other triptans, gepants and non‐specific medication, to offer meaningful contributions to patient care that mimic the real‐world setting.

## CONFLICT OF INTEREST STATEMENT

R.W. reports no conflicts. T.P.J. has received speaker honoraria and/or honoraria as a consultant from AbbVie, Allergan, Grünenthal, Hormosan Pharma, Lilly, Lundbeck, Novartis, Orion Pharma, Pfizer, Sanofi and TEVA. A.R. has received speaker honoraria from Eli‐Lilly, AbbVie, Pfizer, Novartis and Teva and serves as Chief Editor of the Headache and Neurogenic Pain session of *Frontiers in Neurology*. A.J.S. reports personal fees from Invex therapeutics in her role as Director with stock holdings (2019–2023); other for advisory boards from Allergan, Novartis, Cheisi and Amgen outside the submitted work. P.J.G. reports, over the last 36 months, grants from Kallyope and personal fees from Aeon Biopharma, AbbVie, Aurene, CoolTech LLC, Dr Reddy's, Eli‐Lilly and Company, Linpharma, Lundbeck, Pfizer, PureTech Health LLC, Satsuma, Shiratronics, Teva Pharmaceuticals, Tremeau and Vial, and personal fees for advice through Gerson Lehrman Group, Guidepoint, SAI Med Partners, Vector Metric, and fees for educational materials from CME Outfitters and WebMD, and publishing royalties or fees from Massachusetts Medical Society, Oxford University Press, UptoDate and Wolters Kluwer, and a patent magnetic stimulation for headache (No. WO2016090333 A1) assigned to eNeura without fee. JM reports, over the last 36 months, speaker and/or consultant honoraria from Abbvie, Eli‐Lilly, Lundbeck, Orion, Pfizer, TEVA, as well as congress hospitality fees from Abbvie, Dr Reddy’s, GEP Sante, Lundbeck, Parexel and SOS oxygene.

## Supporting information


Data S1.

## Data Availability

As a review, we have no data to share that is not in the tables.
