# Supplementary material for: Sumatriptan‐naproxen sodium in migraine: A review
Source: Eur J Neurol. 2024 Sep 24;31(Suppl 2):e16434. doi: 10.1111/ene.16434 (PMC11422667; doi:10.1111/ene.16434)
Supplement: Supplementary file 1 — Data S1. [file ENE-31-e16434-s001.pdf]

**From:** Gadsden, Michelle
**To:** Purushothaman, Suvetha -; Robinson, Rachel
**Subject:** RE: EJoN-24-1126: SUVEXX Migraine supplement
**Date:** Thursday, July 25, 2024 7:27:00 AM
**Attachments:** image002.png
image003.png
image004.png
image005.png

---

Happy Thursday Suvetha!

Thank you for the update.

I will reach out to Suresh T and the rest of the straive typesetter team today.

Thanks again, much appreciated.

Michelle

**Michelle D. Gadsden**
(she/her/hers)
Associate Project Manager

mgadsden@wiley.com
New Jersey, USA

WILEY

---

**From:** Purushothaman, Suvetha - <spurushoth@wiley.com>
**Sent:** Thursday, July 25, 2024 3:37 AM
**To:** Gadsden, Michelle <mgadsden@wiley.com>; Robinson, Rachel <rrobinso@wiley.com>
**Subject:** Re: EJoN-24-1126: SUVEXX Migraine supplement

Hi Michelle,

We have received the article for production today and I have shared the article ID for your reference: ENE.16434.

Thank you.

Regards,
**Suvetha**
Production Editor

WILEY

---

**From:** Gadsden, Michelle <mgadsden@wiley.com>
**Sent:** 24 July 2024 16:43
**To:** Purushothaman, Suvetha - <spurushoth@wiley.com>; Robinson, Rachel <rrobinso@wiley.com>
**Subject:** RE: EJoN-24-1126: SUVEXX Migraine supplement

Hi Suvetha,

I believe I have it (EJoN-24-1126). Can you confirm?

@Robinson, Rachel

Happy Wednesday Rachel!

I will reach out to Suresh T, Pravin or Ragu of the Straive Typesetter team with the information regarding the 20 pages after the files appear in the EEO system.
It normally takes 24 hours.

Thanks!

Michelle

**Michelle D. Gadsden**
(she/her/hers)
Associate Project Manager

mgadsden@wiley.com
New Jersey, USA

WILEY

---

**From:** Gadsden, Michelle <mgadsden@wiley.com>
**Sent:** Wednesday, July 24, 2024 7:10 AM
**To:** Purushothaman, Suvetha - <spurushoth@wiley.com>; Robinson, Rachel <rrobinso@wiley.com>
**Subject:** RE: EJoN-24-1126: SUVEXX Migraine supplement

Happy Wednesday Suvetha!

Thanks so much.

Can you let me know the editorial article (ScholarOne) number? I can use it to check the EEO system for the export.
I am also familiar with the straive team and I work with them on most of my projects.

Michelle

**Michelle D. Gadsden**
(she/her/hers)
Associate Project Manager

mgadsden@wiley.com
New Jersey, USA

WILEY

---

**From:** Purushothaman, Suvetha - <spurushoth@wiley.com>
**Sent:** Wednesday, July 24, 2024 3:16 AM
**To:** Gadsden, Michelle <mgadsden@wiley.com>; Robinson, Rachel <rrobinso@wiley.com>
**Subject:** Re: EJoN-24-1126: SUVEXX Migraine supplement

Hi Michelle,

Thank you for your email.

I have shared the typesetting contact details below for your reference and you can contact them directly for supplement articles queries.

1-wiley-csr-chn@straive.com

Regards,
**Suvetha**
Production Editor

WILEY

---

**From:** Gadsden, Michelle <mgadsden@wiley.com>
**Sent:** 23 July 2024 20:03
**To:** Robinson, Rachel <rrobinso@wiley.com>; Purushothaman, Suvetha - <spurushoth@wiley.com>
**Subject:** RE: EJoN-24-1126: SUVEXX Migraine supplement

Happy July and Happy Tuesday Suvetha!
It's great to e-meet you.

Please let me know if you are the liaison for this project or will I be provided with a direct contact with the typesetter team?

Please advise and thanks!

Michelle

**Michelle D. Gadsden**
(she/her/hers)
Associate Project Manager

mgadsden@wiley.com
New Jersey, USA

WILEY

---

**From:** Robinson, Rachel <rrobinso@wiley.com>
**Sent:** Tuesday, July 23, 2024 10:14 AM
**To:** Purushothaman, Suvetha - <spurushoth@wiley.com>
**Cc:** Gadsden, Michelle <mgadsden@wiley.com>
**Subject:** FW: EJoN-24-1126: SUVEXX Migraine supplement

Dear Suvetha,

I'd like to introduce you to Michelle Gadsden, who will be the project manager for the supplement article mentioned below by Robert.

This will form the only article within a sponsored supplement, and Michelle will liaise with the sponsors to ensure that we have their approval for the article and the cover before going ahead with publication.

Please could you let Michelle know the WJPCMS article ID once it's been logged in?

Also, please note that we've been asked to limit the extent of the supplement to 20 pages, so that is the maximum page extent for the article. It would be ideal if the typesetters could be made aware of that before starting work on the manuscript, as it might help when they need to make decisions about the size to use for figures and tables.

With best wishes,
Rachel

---

**From:** Huston, Robert <rhuston@wiley.com>
**Sent:** Tuesday, July 23, 2024 10:01 AM
**To:** Losito, Silvana <slosito@wiley.com>; Nadkarni, Aditi <anadkarni@wiley.com>
**Cc:** sommer <sommer@uni-wuerzburg.de>; didier.leys@univ-lille.fr; Purushothaman, Suvetha - <spurushoth@wiley.com>
**Subject:** RE: EJoN-24-1126: SUVEXX Migraine supplement project

Dear all.

Just a quick update on this supplement article.

The review process has just been completed on the revised submission and both reviewers have recommended acceptance.

The article is now with Claudia pending a decision. Once this step has been completed, I will export the files to the production office, advising Suvetha this is a supplement article and should not be assigned to a standard issue.

You may wish to convey further instructions to Suvetha. In which case, please liaise with her directly.

Thank you.

Kind regards,

Robert

---

**From:** Huston, Robert <rhuston@wiley.com>
**Sent:** Wednesday, June 5, 2024 9:07 AM
**To:** Losito, Silvana <slosito@wiley.com>; Nadkarni, Aditi <anadkarni@wiley.com>
**Cc:** sommer <sommer@uni-wuerzburg.de>; didier.leys@univ-lille.fr
**Subject:** EJoN-24-1126: SUVEXX Migraine supplement project

Hello Silvana.

This supplement article has just been submitted and processed. It has now been assigned to Claudia as the acting EiC and Editor and I have flagged it as a supplement article.

Kind regards,
Robert

1 / 18

Awaiting Export | Select Reviewers

Manuscript Information | Audit Trail | Files

**EJoN-24-1126** (REX-PROD-1-C956CE17-866C-4E7F-AE33-D7E4B33479D1-4727D419-7E4C-4B0B-BEE7-DCFDE931B24D-25725)

Submitted: 05-Jun-2024; Last Updated: 05-Jun-2024; In Review: 51min 8sec

- Sumatriptan-Naproxen Sodium in Migraine: A Review
- Goadsby, Peter J. (proxy) (contact); Wilcha, Robyn-Jenia; Afridi, Shazia; Barbanti, Piero; Diener, Hans-Christoph; Jürgens, Tim Patrick; Lanteri-Minet, M; Lucas, Christian; Mawet, Jérôme; Moisset, Xavier; Russo, Antonio; Sacco, Simona; Sinclair, Alexandra; M-L, Sumelahti; tassorelli, cristina
- Review Article
- Revision URL: https://wiley.atyponrex.com/submissionBoard/1/c956ce17-866c-4e7f-ae33-d7e4b33479d1/current
- Wiley - Is for special issue: No edit
- Awaiting Export
- Select Reviewers (Due 15-Jun-2024)
  *0 active selections; 0 invited; 0 agreed; 0 declined; 0 returned*

ED: Sommer, Claudia (proxy)
EIC: Sommer, Claudia (proxy)
TE: Not Assigned
ADM: Rai, Prachi (proxy)
ADM: Excellence, Editorial

HTML | PDF | Supplemental Files | Original Files | Abstract | Cover Letter | External Searches

**Notes** — view all notes

| Note Title | Updated By | Updated On | Edit | Delete |
|---|---|---|---|---|
| For the SUVEXX Migraine supplement project | Excellence Editorial - Managing Editor | **05-Jun-2024 3:54:19 AM** | | |

Add a Note

top

---

**From:** Losito, Silvana <slosito@wiley.com>
**Sent:** Wednesday, June 5, 2024 6:52 AM
**To:** Huston, Robert <rhuston@wiley.com>; Nadkarni, Aditi <anadkarni@wiley.com>
**Subject:** ENE: SUVEXX Migraine supplement project

Hi Robert

I have asked the author to submit the paper and Claudia will be looking after this paper (attached for reference). Let me know if anything else is needed. REX doesn't have this set up as a special issue. @Nadkarni, Aditi for reference.

Best regards
Silvana

**Silvana Losito**
**Senior Publisher**
**Phone**: +44 1865 476516
Oxford, UK

WILEY
